# Genomes comparison of two *Proteus mirabilis* clones showing varied swarming ability

**DOI:** 10.1007/s11033-023-08518-x

**Published:** 2023-05-23

**Authors:** Dawid Gmiter, Ilona Pacak, Sylwia Nawrot, Grzegorz Czerwonka, Wieslaw Kaca

**Affiliations:** Department of Microbiology, Institute of Biology, Faculty of Natural Sciences, Jan Kochanowski University in Kielce, Kielce, Poland

**Keywords:** *Proteus mirabilis*, Draft genome sequence, Swarming motility, Phenotypic heterogeneity

## Abstract

**Background:**

*Proteus mirabilis* is a Gram-negative bacteria most noted for its involvement with catheter-associated urinary tract infections. It is also known for its multicellular migration over solid surfaces, referred to as ‘swarming motility’. Here we analyzed the genomic sequences of two *P. mirabilis* isolates, designated K38 and K39, which exhibit varied swarming ability.

**Methods and results:**

The isolates genomes were sequenced using Illumina NextSeq sequencer, resulting in about 3.94 Mbp, with a GC content of 38.6%, genomes. Genomes were subjected for in silico comparative investigation. We revealed that, despite a difference in swarming motility, the isolates showed high genomic relatedness (up to 100% ANI similarity), suggesting that one of the isolates probably originated from the other.

**Conclusions:**

The genomic sequences will allow us to investigate the mechanism driving this intriguing phenotypic heterogeneity between closely related *P. mirabilis* isolates. Phenotypic heterogeneity is an adaptive strategy of bacterial cells to several environmental pressures. It is also an important factor related to their pathogenesis. Therefore, the availability of these genomic sequences will facilitate studies that focus on the host–pathogen interactions during catheter-associated urinary tract infections.

**Supplementary Information:**

The online version contains supplementary material available at 10.1007/s11033-023-08518-x.

## Introduction

*Proteus mirabilis* is a Gram-negative bacteria most noted for infections of the catheterized urinary tract, known as ‘catheter-associated urinary tract infections’ [1]. It is also known for its remarkable swarming motility. Swarming is multicellular migration over a solid surface, and the swarming motility of *P. mirabilis* has been well studied under laboratory conditions, where cells are allowed to migrate over medium typically supplemented with 1.5%–2.0% agar. Under these conditions, *P. mirabilis* cells undergo dramatic morphological changes, differentiating from swimming cells to highly mobile, hyperflagellated swarming cells, which then return to their classic form after they have spread across the occupied surface (consolidation phase). The repeated phases of cellular differentiation result in the characteristic bull’s-eye pattern observed after incubation [2, 3].

Swarming motility may be responsible for the migration of *P. mirabilis* cells from the urethra upwards into the urinary tract. However, its role in the pathogenesis of *P. mirabilis* cells remains unclear [2, 4].

Further, the pathogenicity of elongated swarming cells is increased by the overexpression of genes responsible for various enzyme activities. Studies have shown higher expression of urease, hemolysin, and ZapA protease by the swarming cells of *P. mirabilis* than for vegetative cells [4]. Moreover, *P. mirabilis* flagellar proteins are recognized by the host immune system, which triggers an inflammatory reaction [4, 5].

Multiple inter- and intracellular factors are involved in the regulation of swarming motility of *P. mirabilis* [2, 3, 6]. However, many aspects of the processes are yet to be fully investigated [6]. Recent studies have demonstrated a natural variation in the swarming ability of *P. mirabilis* strains [7–10], the mechanisms and importance of which in their pathogenesis are not understood. The use of next-generation sequencing (NGS), at both the DNA and RNA levels, may improve our understanding of the genetic mechanisms involved in the regulation of *P. mirabilis* swarming motility. Therefore, in this study, we determined the genomic sequences of two *P. mirabilis* isolates, designated K38 and K39. Despite significant difference in their swarming ability, the isolates showed high genomic similarity, suggesting that one of them probably originated from the other.

## Materials and methods

### Bacterial strains and used genome sequences

*Proteus mirabilis* K38 and K39 were isolated at St Lukas Hospital, Konskie, Poland. The genus and species of the isolates were determined with matrix-assisted laser desorption ionization–time of flight (MALDI-TOF) mass spectrometry, and the isolates were deposited in the Polish Collection of Microorganisms at the Ludwik Hirszfeld Institute of Immunology and Experimental Therapy, Polish Academy of Science, Wroclaw, Poland under PCM numbers 2867 and 2869 for K38 and K39, respectively. The isolates were maintained in lysogeny broth (LB) medium with 8% DMSO at − 80 °C. Isolates K38 and K39 were sent anonymously to our laboratory, and none of the authors had access to any identifying information regarding them. *Proteus mirabilis* genome sequences used in the study were obtained from National Centre of Biotechnological Information (NCBI) and presented in Supplementary Table 1.

### Swarming motility assay and Dienes test

The swarming motility assay was performed as previously described [11], allowing isolates to swarm over LB medium with 1.5% bacteriological agar (swarm agar) for 24 h at 37 °C. The Dienes compatibility of strains was tested according to [9]. In brief, swarm agar was inoculated with 5 µl and 10 µl of a 100-fold dilution of overnight *P. mirabilis* K38 and K39 cultures, respectively, on opposite sides of the plate, and allowed to swarm for 20 h at 37 °C.

### Genomic DNA isolation

The genomic DNA of *P. mirabilis* isolates K38 and K39 was extracted from 1.5 ml of overnight culture with the GenElute™ Bacterial Genomic DNA Kit (Sigma-Aldrich, St. Louis, MO, USA), according to manufacturer’s protocol. The final elution was performed with 100 μl of nuclease-free water. The DNA quality was assessed with a NanoDrop 2000 spectrophotometer (Thermo Fisher Scientific, Waltham, MA, USA).

### Genomes sequencing and de novo assembly

The genomic sequences of *P. mirabilis* K38 and K39 were determined as previously described [12]. Libraries were prepared with the Nextera XT DNA Library Preparation Kit (Illumina Inc., San Diego, CA, USA), according to the manufacturer’s protocol. The libraries were sequenced on the NextSeq system (Illumina) with 2 × 150-bp paired-end reads. The raw reads were trimmed with Trimmomatic v0.39 [13] and their quality was assessed with FASTQC v0.11.9 (http://www.bioinformatics.babraham.ac.uk/projects/fastqc/). Over 91.00% of bases in the sequencing reads had quality scores of 30 (Q30) or higher. The genomes were assembled de novo with Unicycler (Galaxy Version 0.4.8.0, https://usegalaxy.org/ [14], a SPAdes optimizer, as previously described [12]. The default options of SPAdes were selected, including error correction turned on and k-mer in a range of 0.2–0.95 (expressed as a fraction of the read length). Contigs with a fraction of chromosomal depth < 0.25 were filtered out. To optimize Unicycler, the Normal Bridge mode (moderate contig size and moderate misassembly rate) was selected. Contigs shorter than 500 bp were excluded from the final assembly.

### Comparative genomics

After the raw reads were assembled de novo, the contigs obtained were reordered against the reference genome of *P. mirabilis* strain HI4320 with Mauve Contig Mover [15] of the Mauve v2.4.0 software to allow their study [16]. The *P. mirabilis* genomes were then aligned with the Mauve software using the progressiveMauve option [17] and the backbone file was visualized with the R package genoPlotR [18]. Taxonomic affiliation was tested with the FastANI algorithm [19]. For the variant calling analysis, the raw reads were tested with Snippy (Galaxy version 4.4.5 + galaxy2) with the default parameters [20]. Single nucleotide polymorphisms (SNPs) based phylogeny was performed using CSI Phylogeny webserver [21]. The CSI Phylogeny web-server was used with the default options, including minimum depth at SNP positions = 10 × , minimum relative depth at SNP positions = 10%, minimum distance between SNPs = 10 bp, minimum SNP quality = 30, minimum read mapping quality = 25 and minimum Z-score = 1.96. The obtained phylogeny tree includes the *P. mirabilis* HI4320 as a reference genome. The obtained maximum likelihood (ML) phylogenetic tree was midpoint-rooted and visualized using FigTree v1.4.4 (http://tree.bio.ed.ac.uk/software/figtree/). The assembly obtained was primarily functionally annotated with the Rapid Annotation Subsystems Technology (RAST) server using the ClassicRAST annotation scheme, FIGfams version 70, automatic error correction, and automatic frameshift correction [22], and then with the National Center for Biotechnology Information—Prokaryotic Genome Annotation Pipeline (NCBI-PGAP) [23]. Analysis of Kyoto Encyclopedia of Genes and Genomes (KEGG) pathways was conducted by GhostKOALA, an automated metagenome annotation server that characterizes gene functions and pathways based on KEGG Orthology sequence assignments [24]. As an input file, the Amino-Acid FASTA file generated by RAST was used.

### Virulome and resistome

The PathogenFinder v1.1 pathogenicity prediction program (available at https://cge.cbs.dtu.dk/services/PathogenFinder/) was used to examine the likelihood that *P. mirabilis* K38 and K39 were human pathogens [25]. The presence of genes related to the most important virulence factors was also tested, as previously described [12], using an in-house local database of virulence genes created with the makeblastdb option of BLAST + [26]. The genes were selected based on the previously described genome of *P. mirabilis* HI4320 [27]. The databases included genes responsible for ureolitic, proteolitic and hemolytic activity, motility (flagellum synthesis and chemotaxis), and fimbriae synthesis.

Bacterial antimicrobial resistance was predicted with Resistance Gene Identifier (RGI) based on the Comprehensive Antibiotic Resistance Database (CARD) [28]. The selection criteria were perfect and strict hits only, and nudges above 95% were excluded. The sequence quality was defined as high coverage of the DNA template.

## Results

### Characterization of *Proteus mirabilis* isolates

**Fig. 1:**
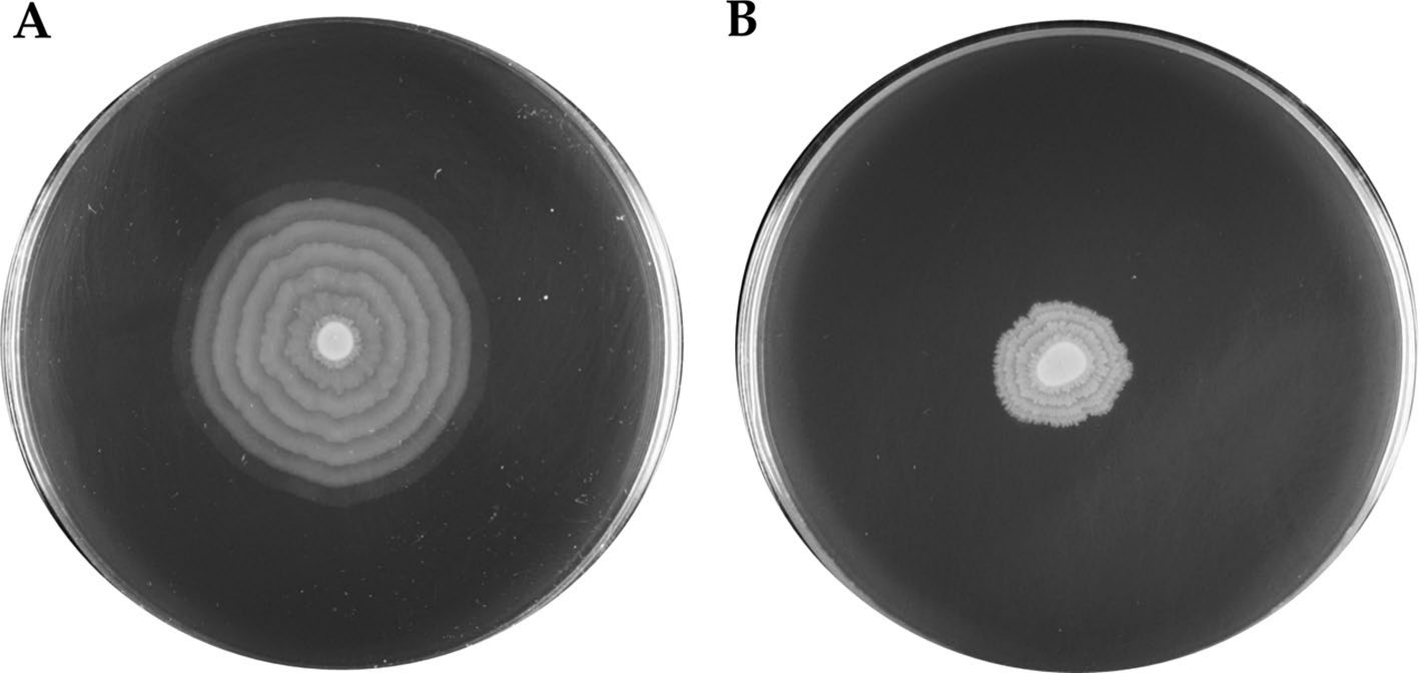
Swarming motility of *Proteus mirabilis* isolates K38 (**A**) and K39 (**B**) on LB agar plates after 20 h of incubation

*Proteus mirabilis* isolates K38 and K39 were distinguishable by differences in their swarming motility. *Proteus mirabilis* K38 showed the characteristic pattern of swarming over agar medium, whereas K39 showed restricted swarming. After incubation on LB medium supplemented with 1.5% agar for 20 h, the mean colony diameter of the K38 was 5.0 ± 1.0 cm and that of K39 was 1.8 ± 0.3 cm. Figure 1 shows representative images of both isolate swarms. The K38 isolate presents the classic bull’s-eye pattern of swarming, characteristic of *P. mirabilis*. The zones of migration and consolidation are clearly visible and easy to distinguish. The edge of each zone is smooth. However, the K39 isolate is characterized by a disturbed pattern of swarming, clearly different from that of K38 and most other *P. mirabilis* strains. Interestingly, when subjected to the Dienes test, the K38 and K39 isolates did not form a Dienes line, indicating their relatedness [29].

### General characteristic of K38 and K39 genome sequences

The genomic DNA of K38 and K39 were sequenced with the Illumina NextSeq system with 305-fold coverage for both. The raw reads were assembled de novo with Unicycler into 53 and 54 contigs for K38 and K39, respectively. Table 1 presents the basic characteristics of the analyzed genomic sequences, determined with the RAST server. The estimated sizes of the draft genomes of the two isolates were about 3.94 Mbp, with a GC content of 38.6%. The difference in the genome lengths was only 389 bp. RAST also provided the basic statistics for the quality of the genome assemblies. Among these, N50 is a metric widely used to assess the contiguity of an assembly, and is defined as the length of the shortest contig for which longer and equal length contigs cover at least 50% of the assembly [30].

**Table 1:** Genomes assembly statistics based on RAST annotation server

Attribute	Value	
	K38	K39
Genome size (bp)	3,942,802	3,943,189
%GC	38.6	38.6
N50 (bp)	259800	259800
L50 (bp)	6	6
Number of contigs (with PEGs)	53	54
Number of subsystems	498	498
Number of coding sequences	3568	3567
Number of RNAs	80	80
Number of plasmids	0	0

An overview of the subsystem coverage and subsystem feature counts predicted for the *P. mirabilis* K38 and K39 isolates with the RAST server are presented in Supplementary Table 2. Both genomic sequences were submitted to the National Center for Biotechnology Information (NCBI) GenBank database and annotated with the NCBI–PGAP. The values obtained are consistent with those previously reported for both complete and draft *P. mirabilis* genomic sequences [9, 12, 27, 31–33].

Further characteristics of the genomic sequences of isolates K38 and K39 were determined with annotation in a KEGG pathway analysis with the GhostKoala online application. In total, 2353 entries were annotated in the genomes of K38 and K39. The distribution and frequencies of KEGG pathway annotations are presented in Supplementary Table 3.

**Fig. 2:**
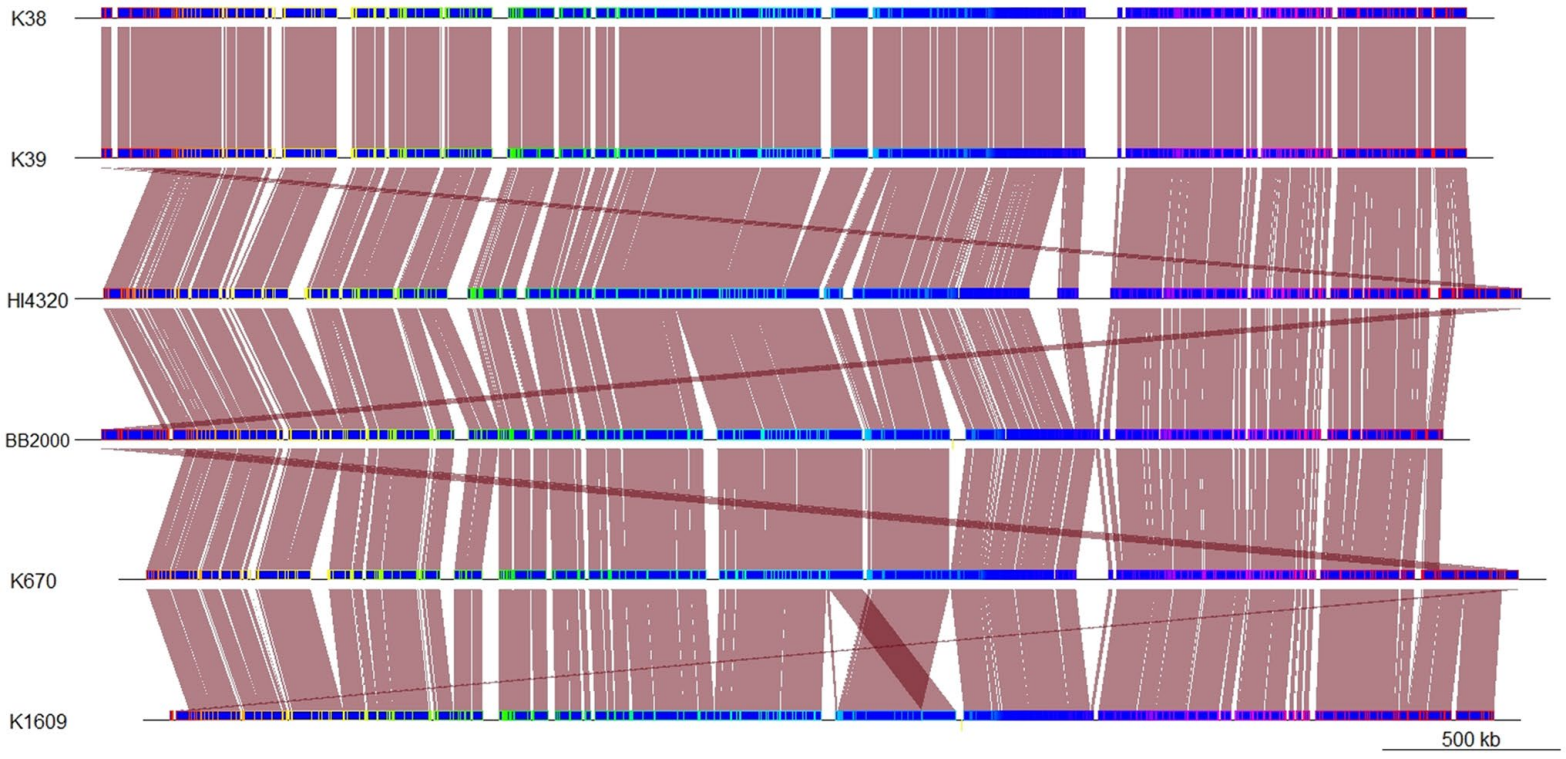
Whole genome comparison of selected *Proteus mirabilis* genomes using progressiveMauve option of Mauve software. Genomes are represented in blue, and blocks with borders of different colors are homologous between genomes The backbone file was visualized using the R package genoPlotR

**Fig. 3:**
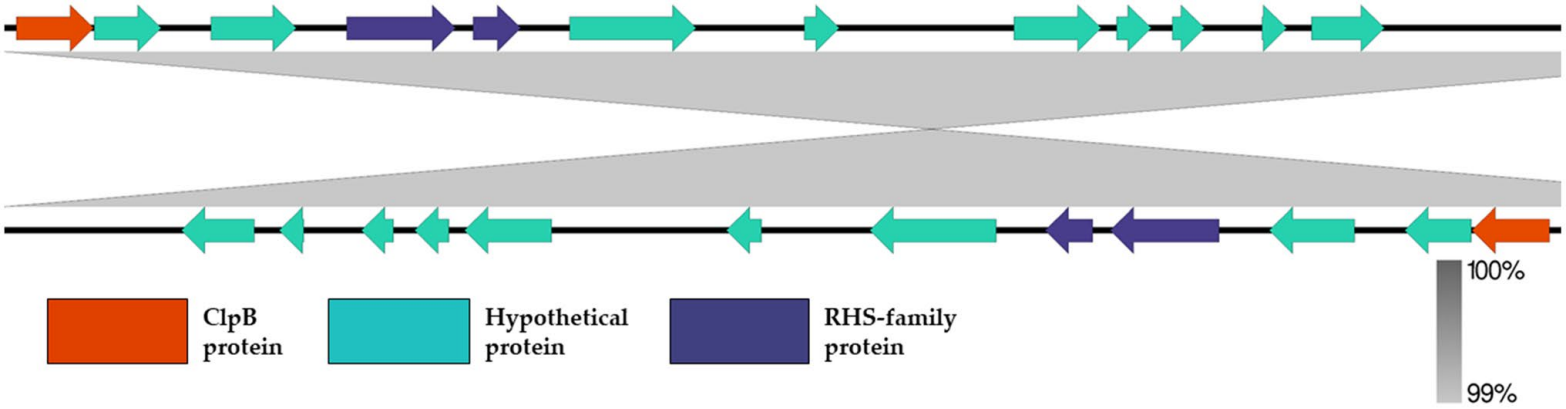
Comparison of conting 28 from studied *Proteus mirabilis* K38 (top) and K39 (bottom) isolates

The *P. mirabilis* genomes were visually compared with the progressiveMauve option in the Mauve software (Fig. 2). Genomic rearrangement events are shown by intersecting lines that link locally collinear blocks (LCBs). The LCBs were calculated with Mauve to identify conserved segments that appear to be internally free of genomic rearrangements. Closer examination revealed that the genomes of isolates K38 and K39 share a high level of similarity in terms of their sequence organization. However, a small 7532-bp region, constituting contig 28, appears to be inverted between the genomes. The RAST server annotated 12 open read frames within this region, encoding nine hypothetical proteins, two rearrangement hotspot (RHS) family proteins, and the ClpB protein (Fig. 3).

### Phylogenetic analysis

The average nucleotide identity (ANI) is a metric frequently used to describe the phylogenomic relationships between bacterial strains [34]. We tested the percentage similarity between the two studied isolates and the reference HI4320 genome, using FastANI. The results are presented in Table 2. *Proteus mirabilis* K38 and K39 share up to 100% similarity, defined with the ANI value. The differences between them result from the differences in their genome lengths, and depend upon which is used as the reference genome in the analysis. However, both isolates show the same similarity to HI4320. The observed ANI value between studied isolates and reference genome of HI4320 far exceeds the generally accepted 95% cut-off level for the taxonomic affiliation of newly sequenced genomes [34].

**Table 2:** The Average nucleotide identity (ANI) comparison of *Proteus mirabilis* K38, K39 and HI4320 genome sequences

Reference genome	Query genome	ANI Estimate (%)
HI4320	K38	98,97
	K39	98,97
K38	K39	99,99
K39	K38	100

A further simple variant calling analysis was performed with Snippy, in which the HI4320 genomic sequence was used to test for the presence of different types of variants in the raw reads of the K38 and K39 genomes. A similar K38 vs K39 comparison was performed, using the assembled contigs of both isolates against their raw reads. In this second analysis, no differences were observed when the genomes were compared. Over 20,000 variants were detected between HI4320 and both K38 and K39. The FILTERs status of all variants was PASS, indicating that the variants in the raw data were true calls and not false positives resulting from low coverage. The variants were categorized as complex deletions and insertions (in/dels) and single- (SNPs) and multiple-nucleotide polymorphisms (MNPs) (Table 3). This observation is consistent with the results of a previous study [12].

**Table 3:** Results of variant calling analysis between studied *Proteus mirabilis* isolates and reference strain HI4320

Strain	All	Detected variants				
		Complex	Del	Ins	SNPs	MNPs
K38	20340	1410	167	165	18325	273
K39	20370	1420	169	166	18341	274

**Fig. 4:**
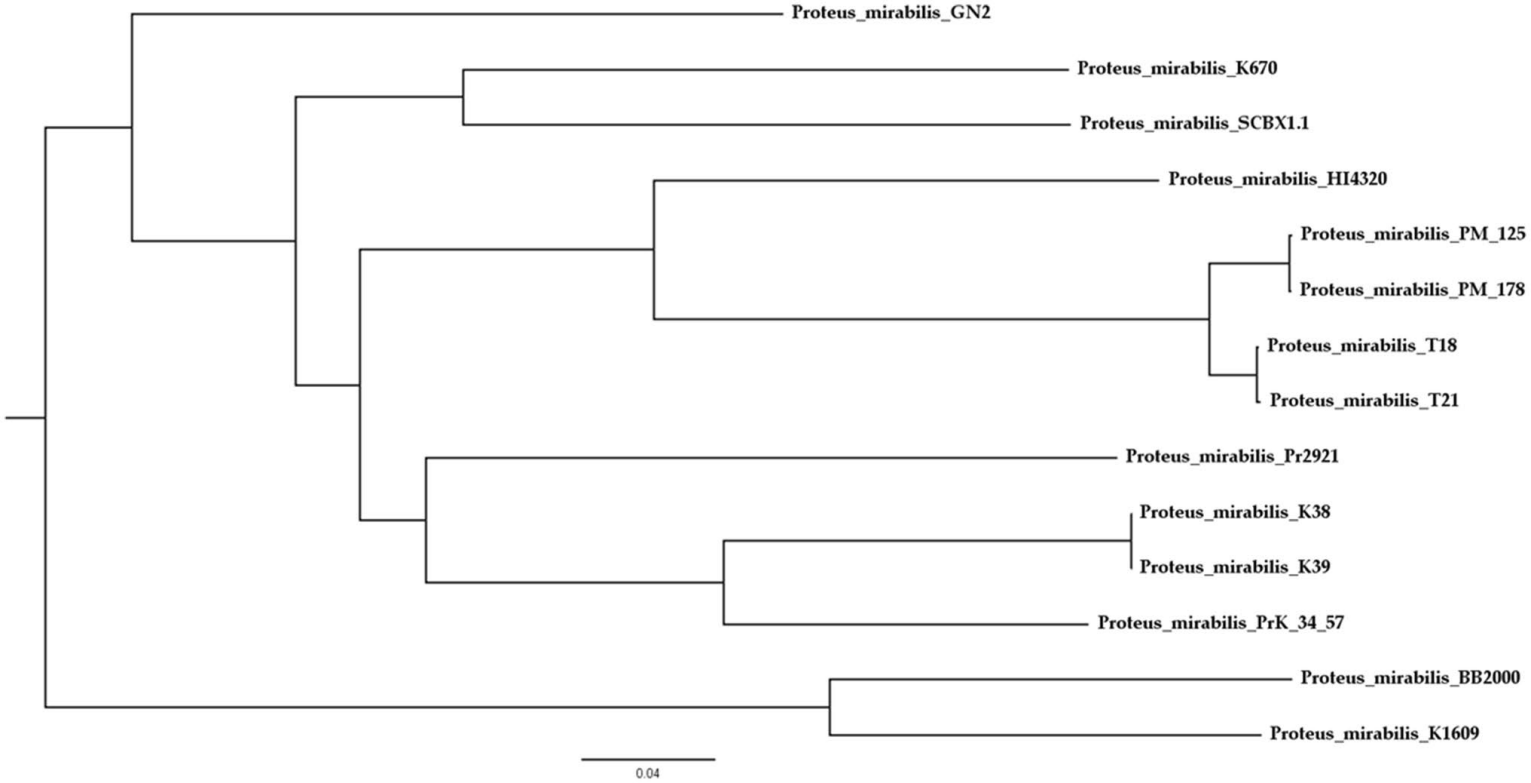
SNPs-based phylogeny of studied *Proteus mirabilis* isolated with reference genomes of *Proteus mirabilis* from GenBank. The phylogenetic tree was obtained using CSI Phylogeny, midpoint-rooted and visualized using FigTree

The SNP-based phylogenetic relationships between the studied isolates and selected *P. mirabilis* genomic sequences downloaded from GenBank were determined with the online application CSI Phylogeny. The genomic sequence of *P. mirabilis* strain HI4320 was used as the reference. The percentage of the reference genome covered by all the isolates was 82.67%, and 4,063,606 positions were found in all the genomes analyzed. Based on the detected SNPs, a maximum likelihood (ML) phylogenetic tree was constructed (Fig. 4). The tree confirmed that isolates K38 and K39 share high genetic similarity. The SNP counts (Table 4) showed that isolates shared the same genetic background, and no SNP distinguished the two isolates within the region compared. The studied isolates shared greatest genetic similarity with strain PrK 34/57, the genome of which was previously described by us [12]. These results indicated that the isolates were more closely related to the reference strain HI4320 than to BB2000, another frequently studied *P. mirabilis* strain [33]. We previously noted that *P. mirabilis* strains PM_125 and PM_178 also seem to be clones [9], sharing an average nucleotide identity of 100% [35]. However, CSI Phylogeny identified 44 SNPs between these strains. Similarly, strains T18 and T21 showed genetic relatedness, with 74 SNPs, and were identified as clones with restricted swarming ability [36].

**Table 4:** Count of single nucleotide polymorphisms (SNPs) identified between selected *Proteus mirabilis* genome sequences using CSI Phylogeny

Strain	BB2000	GN2	K1609	K38	K39	K670	PM_125	PM_178	Pr2921	PrK 34/57	SCBX1.1	T18	T21	HI4320
BB2000	0	18112	9985	19608	19608	19542	19966	19948	19819	19416	19297	19729	19699	18544
GN2	18112	0	17886	15025	15025	14489	16596	16580	14749	14517	14114	16356	16340	15449
K1609	9985	17886	0	19397	19397	19422	19719	19703	19544	19229	19123	19490	19455	18420
K38	19608	15025	19397	0	0	14381	15093	15079	13655	8872	14383	14769	14758	14287
K39	19608	15025	19397	0	0	14381	15093	15079	13655	8872	14383	14769	14758	14287
K670	19542	14489	19422	14381	14381	0	15998	15977	14436	13577	12779	15703	15685	15049
PM_125	19966	16596	19719	15093	15093	15998	0	44	15506	15198	16080	1755	1803	12121
PM_178	19948	16580	19703	15079	15079	15977	44	0	15486	15186	16064	1733	1781	12107
Pr2921	19819	14749	19544	13655	13655	14436	15506	15486	0	13435	14300	15240	15218	14427
PrK 34/57	19416	14517	19229	8872	8872	13577	15198	15186	13435	0	14034	14857	14839	14349
SCBX1.1	19297	14114	19123	14383	14383	12779	16080	16064	14300	14034	0	15817	15795	15145
T18	19729	16356	19490	14769	14769	15703	1755	1733	15240	14857	15817	0	74	11838
T21	19699	16340	19455	14758	14758	15685	1803	1781	15218	14839	15795	74	0	11788
HI4320	18544	15449	18420	14287	14287	15049	12121	12107	14427	14349	15145	11838	11788	0

### Swarming motility related genes

Because isolates K38 and K39 differ greatly in their swarming ability, we focused on the annotation and comparison of the genes involved in this process. As previously described, all flagellar and chemotaxis-related genes are located within a single 53.3-kb locus in the HI4320 genome [27]. Similar organization of the swarming-related genes was observed in the genomic sequences of isolates K38 and K39. These genes were found within contig 2 of the assemblies generated, sharing 100% similarity between K38 and K39 and 95.45%–100% similarity with the reference HI4320 genome (Supplementary Table 4).

### Virulome and resistome identification

The pathogenicity of isolates K38 and K39 was examined with PathogenFinder v1.1, a pathogenicity prediction program. Both were predicted to be human pathogens (with a probability of 0.788) (Supplementary Table 5). An additional analysis using a previously generated local BLAST + database consisting of virulence-related genes previously annotated in strain HI4320 [27] showed that both isolates K38 and K39 contained a complete set of genes for the most important virulence factors known in *P. mirabilis*. Further, homologues of genes responsible for resistance to aminoglycosides, beta-lactams, fluoroquinolones, macrolides, and phenicol were identified consistently in both strains with the RGI tool, and are presented in Supplementary Table 6.

## Discussion

In this report we presented an example of two *P. mirabilis* isolates, named K38 and K39, that differ in swarming ability. Analysis of the genomic sequences of isolates indicated their clonality, which was also indicated by the Dienes test. The Dienes phenomenon involves the formation of the so-called ‘Dienes line’ between the swarming colonies of unrelated strains of *P. mirabilis*. Earlier studies have demonstrated a relationship between genetic similarity and the relatedness detected with the Dienes test [37]. This finding suggested that one or other of isolates K38 and K39 originated from the other. The difference in their ability to migrate over solid medium, despite the genetic similarity detected with the Dienes test, was surprising. Previously, Drzewiecka et al. [29] described two isolates of *P. mirabilis*, designated 3 B-m and 3 B-k, which were isolated from urine and feces of a hospitalized patient in Poland [29]. These isolates showed relatedness on the Dienes test and significant genetic similarity with genotyping methods. Other studies have also presented genomes of *P. mirabilis* isolates that were clones (T18 and T21; PM_125 and PM_178) [35, 36]. However, our analysis with CSI Phylogeny indicated a higher degree of identity between the K39 and K39 genomes than between the clonal strains mentioned above. Importantly, *P. mirabilis* clonal isolates T18 and T21 were described as strains with limited swarming ability [36]. Other studies have compared strains with different swarming abilities [7–10], but unlike K38 and K39, they showed significant genetic distances to each other.

Further analyses indicated the high pathogenic potential of isolates K39 and K39. Numerous genes encoding proteins that can act as virulence factors and genes conferring antibiotic resistance were identified in their genomes. These results are consistent with previous studies [11, 12, 32] and the nature of *P. mirabilis*, an opportunistic pathogen.

By considering the factors that regulate swarming motility [2, 3, 6], a hypothesis can be proposed about the genetic diversity of the isolates to explain their different phenotypes. However, the comparative analysis performed identified no differences between the isolates that could explain the observed phenomenon. This included the lack of SNPs detected in a phylogenetic analysis and a detailed analysis of the region containing the genes associated with swarming motility.

Despite the lack of differences in the nucleotide sequences of the K39 and K39 genomes, a comparative analysis revealed a small region of DNA inversion between these genomes. Within this region, 12 open reading frames were identified, predominantly encoding small proteins of unknown function. Interestingly, this region also contains the gene encoding the ClpB protein, a key chaperone that plays a crucial role in bacterial survival under various forms of stress, particularly heat shock, via its disaggregase activity. It has recently been reported that ClpB also regulates the secretion of bacterial effector molecules related to the type VI secretion systems [38].

The lack of mutations in sequences encoding the proteins associated with swarming motility suggests that the different phenotypes of K39 and K39 might result from different gene expression profiles [39]. However, it is very likely that the promoter sequences regulating the level of gene expression are also identical in the isolates. In this context, the significance of the identified inversion is puzzling. The reorganization of the genomic structure may lead to a different gene expression profile, which may affect the variability of the phenotype [40]. However, this hypothesis requires further analysis.

The difference in the swarming abilities of K38 and K39 may be an example of phenotypic heterogeneity—functional diversity among genetically identical cells. This phenomenon is often the result of an interaction between bacterial cells and the growth environment, and is a way to adapt to a changing environment [41–43]. In the context of bacterial infections, this phenomenon is important for the virulence of strains [43]. In the case of *P. mirabilis*, the phenomenon of phenotypic heterogeneity driven by phase variation may include the formation of a hyper-swarming mutant resulting from the expression of hybrid FlaAB flagella [44].

Our results not only raise questions about the mechanism underlying the phenotypic variability between *P. mirabilis* strains K38 and K39, but also about its importance in their pathogenesis. Swarming motility is considered to be a virulence factor of *P. mirabilis*. It potentially allows cells to migrate along the surface of the catheter [4]. Swarming cells are also observed in biofilms formed in artificial urine [45]. On the other hand, strains lacking the capacity to swarm are capable of forming a crystalline biofilm [46]. The expression of enzymes important for the virulence of *P. mirabilis* (urease, proteases, and hemolysin) is increased during swarming migration [4]. However, research has shown that strains with a limited swarming ability might show greater expression of the *zapA* gene [10]. The *zapA* gene encodes an extracellular metalloprotease that hydrolyzes a wide range of protein and peptide substrates, including immune system proteins [47, 48]. It has also been reported that strains with different swarming abilities activate different apoptosis pathways, as demonstrated in a normal human prostate epithelial cell model [8]. Moreover, flagellin, the structural component of the flagella, induced the expression of proinflammatory chemokines in T24 bladder cell cultures and in the mouse bladder after instillation [49]. For this reason, reducing the amount of flagellin, essential for swarming motility, may be a way to avoid the immune response induced by *P. mirabilis* K39 cells. However, this hypothesis requires further research.

## Conclusions

In this study, we characterized and comparatively analyzed the genomic sequences of two *P. mirabilis* isolates, which despite their high clonality, displayed different swarming motility. We showed that phenotypic diversity does not only occur in genetically distinct strains of *P. mirabilis*, but also in closely related strains. The high genetic similarity between the isolates raised questions about the mechanism underlying the observed phenotypic variation. It is possible that this variability is the result of the adaptation of *P. mirabilis* to the colonization of its host. It is worth asking what molecular signal could lead to the observed phenomenon and how it is important to the pathogenesis of *P. mirabilis*. Answering these questions may clarify the host–pathogen interactions during urinary tract infections. The genomic sequences of *P. mirabilis* isolates K38 and K39 should provide a basis for further research to explain the observed phenomenon.

The Whole Genome Shotgun Projects of *P. mirabilis* isolates K38 and K39 have been deposited at GenBank (http://www.ncbi.nlm.nih.gov) under the BioProject ID PRJNA506729.

## Supplementary Information

Below is the link to the electronic supplementary material.

Supplementary file1 (DOCX 14 KB)

Supplementary file2 (DOCX 16 KB)

Supplementary file3 (DOCX 15 KB)

Supplementary file4 (DOCX 24 KB)

Supplementary file5 (DOCX 14 KB)

Supplementary file6 (DOCX 17 KB)

## Data Availability

Data that support the findings of this study are included in this published article (and its supplementary information files). The detailed datasets about genome annotation generated during and/or analysed during the current study are available from the corresponding author on reasonable request.
