# Supplementary material for: Genomes comparison of two Proteus mirabilis clones showing varied swarming ability
Source: Mol Biol Rep. 2023 May 23;50(7):5817–26. doi: 10.1007/s11033-023-08518-x (PMC10290045; doi:10.1007/s11033-023-08518-x)
Supplement: Supplementary file 1 — Supplementary file1 (DOCX 14 KB) [file 11033_2023_8518_MOESM1_ESM.docx]

**Table S1** *Proteus mirabilis* genomes used in the study.

| Strain | Accession number | Genome size (bp) |
| --- | --- | --- |
| HI4320 | AM942759 | 4,063,606 |
| BB2000 | [CP004022](https://www.ncbi.nlm.nih.gov/nuccore/CP004022) | 3,846,754 |
| GN2 | CP026581 | 4,012,640 |
| K1609 | [CP028522](https://www.ncbi.nlm.nih.gov/nuccore/CP028522) | 3,817,795 |
| K670 | [CP028356](https://www.ncbi.nlm.nih.gov/nuccore/CP028356) | 3,935,626 |
| Pr2921 | LGTA00000000 | 3,924,499 |
| PM_125 | LWUL00000000 | 3,955,474 |
| PM_178 | LWUM00000000 | 3,969,065 |
| PrK 34/57 | JAAMPE000000000 | 3,970,593 |
| T18 | CP017085 | 4,131,426 |
| T21 | CP017082 | 4,090,879 |
| SCBX1.1 | CP047112 | 4,200,651 |
