# Supplementary material for: Genomes comparison of two Proteus mirabilis clones showing varied swarming ability
Source: Mol Biol Rep. 2023 May 23;50(7):5817–26. doi: 10.1007/s11033-023-08518-x (PMC10290045; doi:10.1007/s11033-023-08518-x)
Supplement: Supplementary file 2 — Supplementary file2 (DOCX 16 KB) [file 11033_2023_8518_MOESM2_ESM.docx]

**Table S2** Subsystems distribution of *Proteus mirabilis* K38 and K39 isolates based on RAST annotation server.

| ***Proteus mirabilis* isolate** | **K38** | **K39** |
| --- | --- | --- |
| **Subsystem Coverage** | | |
| In Subsystem | 56% | 56% |
| Not in Subsystem | 44% | 44% |
| **Subsystems Feature Counts** | | |
| Cofactors, vitamins, prosthetic groups, pigments | 249 | 248 |
| Cell wall and capsule | 162 | 162 |
| Virulence, disease, and defence | 71 | 71 |
| Potassium metabolism | 25 | 25 |
| Photosynthesis | 0 | 0 |
| Miscellaneous | 43 | 43 |
| Phages, prophages, transposable elements, plasmids | 27 | 27 |
| Membrane transport | 184 | 184 |
| Iron acquisition and metabolism | 58 | 58 |
| RNA metabolism | 225 | 225 |
| Nucleosides and nucleotides | 97 | 97 |
| Protein metabolism | 272 | 272 |
| Cell division and cell cycle | 38 | 38 |
| Motility and chemotaxis | 57 | 57 |
| Regulation and cell signaling | 97 | 97 |
| Secondary metabolism | 4 | 4 |
| DNA metabolism | 106 | 106 |
| Fatty acids, lipids, and isoprenoids | 113 | 113 |
| Nitrogen metabolism | 27 | 27 |
| Dormancy and sporulation | 6 | 6 |
| Respiration | 152 | 152 |
| Stress response | 134 | 134 |
| Metabolism of aromatic compounds | 3 | 3 |
| Amino acids and derivatives | 370 | 370 |
| Sulfur metabolism | 37 | 37 |
| Phosphorus metabolism | 35 | 35 |
| Carbohydrates | 331 | 331 |
|  |  |  |
|  |  |  |
