# Supplementary material for: Genomes comparison of two Proteus mirabilis clones showing varied swarming ability
Source: Mol Biol Rep. 2023 May 23;50(7):5817–26. doi: 10.1007/s11033-023-08518-x (PMC10290045; doi:10.1007/s11033-023-08518-x)
Supplement: Supplementary file 3 — Supplementary file3 (DOCX 15 KB) [file 11033_2023_8518_MOESM3_ESM.docx]

**Table S3** KEGG pathway analysis of *Proteus mirabilis* K38 and K39 isolates. Proteins were identified and categorized using the GhostKOALA tool against the Amino-Acid file generated by RAST server.

|  | K38 | K39 |
| --- | --- | --- |
| Protein families: genetic information processing | 335 | 336 |
| Protein families: signaling and cellular processing | 293 | 293 |
| Environmental information processing | 211 | 210 |
| Carbohydrate metabolism | 198 | 198 |
| Genetic information processing | 178 | 178 |
| Unclassified: metabolism | 150 | 150 |
| Unclassified | 126 | 126 |
| Metabolism of cofactors and vitamins | 120 | 120 |
| Amino acid metabolism | 108 | 108 |
| Energy metabolism | 95 | 95 |
| Nucleotide metabolism | 82 | 82 |
| Cellular processes | 73 | 73 |
| Unclassified: signeling and cellular processes | 72 | 72 |
| Protein families: metabolism | 67 | 67 |
| Unclassified: genetic information processing | 54 | 54 |
| Lipid metabolism | 53 | 53 |
| Glycan biosynthesis and metabolism | 52 | 52 |
| Humas diseases | 41 | 41 |
| Metabolism of other amino acids | 25 | 25 |
| Metabolis of terpenoids and polyketides | 13 | 13 |
| Organismal systems | 3 | 3 |
| Xenobiitics degradation and metabolism | 3 | 3 |
| Biosynthesis of other secondary metabolites | 1 | 1 |
|  | 2353 | 2353 |
