# Supplementary material for: Genomes comparison of two Proteus mirabilis clones showing varied swarming ability
Source: Mol Biol Rep. 2023 May 23;50(7):5817–26. doi: 10.1007/s11033-023-08518-x (PMC10290045; doi:10.1007/s11033-023-08518-x)
Supplement: Supplementary file 4 — Supplementary file4 (DOCX 24 KB) [file 11033_2023_8518_MOESM4_ESM.docx]

**Table S4** Comparison of swarming related genes between isolates *Proteus mirabilis* isolates 38 and K39 and reference strain HI4320.

| Contig | Gene | Percentage of identical matches (%) | Alignment length | Number of mismatches | Number of gap openings | Start of alignment in query | End of alignment in query | Start of alignment in subject | End of alignment in subject | Expectation value (E-value) | Bit score |
| --- | --- | --- | --- | --- | --- | --- | --- | --- | --- | --- | --- |
| 2 | *fliZ* | 99.81 | 531 | 1 | 0 | 194496 | 195026 | 1 | 531 | 0.0 | 954 |
| 2 | *fliA* | 100.00 | 723 | 0 | 0 | 195085 | 195807 | 1 | 723 | 0.0 | 1305 |
| 2 | *flaB* | 98.28 | 1104 | 16 | 1 | 196250 | 197350 | 1 | 1104 | 0.0 | 1904 |
| 2 | *flaA* | 95.45 | 1098 | 44 | 2 | 197660 | 198757 | 1 | 1092 | 0.0 | 1752 |
| 2 | *flaD/fliD* | 97.46 | 1419 | 36 | 0 | 199144 | 200562 | 1 | 1419 | 0.0 | 2397 |
| 2 | *fliS* | 99.50 | 399 | 2 | 0 | 200588 | 200986 | 1 | 399 | 0.0 | 711 |
| 2 | *fliT* | 99.72 | 354 | 1 | 0 | 201005 | 201358 | 1 | 354 | 0.0 | 635 |
| 2 | *fliE* | 99.68 | 312 | 1 | 0 | 205315 | 205626 | 1 | 312 | 9.00e-159 | 559 |
| 2 | *fliF* | 99.71 | 1722 | 5 | 0 | 205903 | 207624 | 1 | 1722 | 0.0 | 3084 |
| 2 | *fliG* | 99.90 | 999 | 1 | 0 | 207621 | 208619 | 1 | 999 | 0.0 | 1798 |
| 2 | *fliH* | 99.31 | 723 | 5 | 0 | 208612 | 209334 | 1 | 723 | 0.0 | 1282 |
| 2 | *fliI* | 99.85 | 1374 | 2 | 0 | 209334 | 210707 | 1 | 1374 | 0.0 | 2470 |
| 2 | *fliJ* | 100.00 | 447 | 0 | 0 | 210738 | 211184 | 1 | 447 | 0.0 | 807 |
| 2 | *fliK* | 99.51 | 1413 | 7 | 0 | 211184 | 212596 | 1 | 1413 | 0.0 | 2517 |
| 2 | *fliL* | 100.00 | 483 | 0 | 0 | 212774 | 213256 | 1 | 483 | 0.0 | 872 |
| 2 | *fliM* | 99.81 | 1032 | 2 | 0 | 213262 | 214293 | 1 | 1032 | 0.0 | 1853 |
| 2 | *fliN* | 100.00 | 411 | 0 | 0 | 214286 | 214696 | 1 | 411 | 0.0 | 742 |
| 2 | *fliO* | 99.78 | 447 | 1 | 0 | 214700 | 215146 | 1 | 447 | 0.0 | 802 |
| 2 | *fliP* | 99.74 | 771 | 2 | 0 | 215146 | 215916 | 1 | 771 | 0.0 | 1382 |
| 2 | *fliQ* | 100.00 | 270 | 0 | 0 | 215931 | 216200 | 1 | 270 | 1.34e-137 | 488 |
| 2 | *fliR* | 99.23 | 783 | 6 | 0 | 216206 | 216988 | 1 | 783 | 0.0 | 1386 |
| 2 | *flgL* | 100.00 | 945 | 0 | 0 | 217301 | 218245 | 1 | 945 | 0.0 | 1705 |
| 2 | *flgK* | 99.39 | 1644 | 10 | 0 | 218271 | 219914 | 1 | 1644 | 0.0 | 2920 |
| 2 | *flgJ* | 99.29 | 987 | 7 | 0 | 220033 | 221019 | 1 | 987 | 0.0 | 1749 |
| 2 | *flgI* | 99.82 | 1107 | 2 | 0 | 221019 | 222125 | 1 | 1107 | 0.0 | 1988 |
| 2 | *flgH* | 99.73 | 744 | 2 | 0 | 222140 | 222883 | 1 | 744 | 0.0 | 1333 |
| 2 | *flgG* | 99.49 | 783 | 4 | 0 | 222946 | 223728 | 1 | 783 | 0.0 | 1395 |
| 2 | *flgF* | 99.34 | 756 | 5 | 0 | 223748 | 224503 | 1 | 756 | 0.0 | 1342 |
| 2 | *flgE* | 99.84 | 1221 | 2 | 0 | 224525 | 225745 | 1 | 1221 | 0.0 | 2194 |
| 2 | *flgD* | 97.88 | 801 | 11 | 1 | 225774 | 226574 | 1 | 795 | 0.0 | 1370 |
| 2 | *flgC* | 100.00 | 405 | 0 | 0 | 226587 | 226991 | 1 | 405 | 0.0 | 731 |
| 2 | *flgB* | 99.28 | 414 | 3 | 0 | 226997 | 227410 | 1 | 414 | 0.0 | 734 |
| 2 | *flgA* | 99.70 | 657 | 2 | 0 | 227620 | 228276 | 1 | 657 | 0.0 | 1177 |
| 2 | *flgM* | 99.33 | 300 | 2 | 0 | 228703 | 229002 | 1 | 300 | 3.58e-151 | 533 |
| 2 | *flgN* | 99.55 | 441 | 2 | 0 | 229014 | 229454 | 1 | 441 | 0.0 | 787 |
| 2 | *floA* | 95.47 | 1257 | 57 | 0 | 229605 | 230861 | 1 | 1257 | 0.0 | 2011 |
| 2 | *flhA* | 99.04 | 2091 | 20 | 0 | 231273 | 233363 | 1 | 2091 | 0.0 | 3681 |
| 2 | *flhB* | 99.48 | 1149 | 6 | 0 | 233356 | 234504 | 1 | 1149 | 0.0 | 2046 |
| 2 | *cheZ* | 99.70 | 657 | 2 | 0 | 237333 | 237989 | 1 | 657 | 0.0 | 1177 |
| 2 | *cheY* | 100.00 | 390 | 0 | 0 | 237999 | 238388 | 1 | 390 | 0.0 | 704 |
| 2 | *cheB* | 99.05 | 1053 | 10 | 0 | 238448 | 239500 | 1 | 1053 | 0.0 | 1855 |
| 2 | *cheR* | 99.66 | 891 | 3 | 0 | 239493 | 240383 | 1 | 891 | 0.0 | 1594 |
| 2 | *tap* | 99.82 | 1647 | 3 | 0 | 240390 | 242036 | 1 | 1647 | 0.0 | 2957 |
| 2 | *cheD* | 99.65 | 1707 | 6 | 0 | 242096 | 243802 | 1 | 1707 | 0.0 | 3052 |
| 2 | *cheW* | 100.00 | 500 | 0 | 0 | 244150 | 244649 | 1 | 500 | 0.0 | 902 |
| 2 | *cheA* | 98.92 | 2226 | 18 | 1 | 244672 | 246897 | 1 | 2220 | 0.0 | 3908 |
| 2 | *motB* | 99.91 | 1050 | 1 | 0 | 246933 | 247982 | 1 | 1050 | 0.0 | 1890 |
| 2 | *motA* | 99.89 | 894 | 1 | 0 | 247985 | 248878 | 1 | 894 | 0.0 | 1608 |
| 2 | *flhC* | 99.83 | 582 | 1 | 0 | 249013 | 249594 | 1 | 582 | 0.0 | 1046 |
| 2 | *flhD* | 100.00 | 351 | 0 | 0 | 249597 | 249947 | 1 | 351 | 0.0 | 634 |
