# Supplementary material for: Genomes comparison of two Proteus mirabilis clones showing varied swarming ability
Source: Mol Biol Rep. 2023 May 23;50(7):5817–26. doi: 10.1007/s11033-023-08518-x (PMC10290045; doi:10.1007/s11033-023-08518-x)
Supplement: Supplementary file 5 — Supplementary file5 (DOCX 14 KB) [file 11033_2023_8518_MOESM5_ESM.docx]

**Table S5** Pathogenic probability of *Proteus mirabilis* K38 and K39 based on PathogenFinder analysis.

| Strain | K38 | K39 |
| --- | --- | --- |
| Probability of being a human pathogen | 0.788 | 0.788 |
| Input proteome coverage (%) | 6.15 | 6.14 |
| Matched Pathogenic Families | 185 | 185 |
| Matched Not Pathogenic Families | 33 | 33 |
