# Supplementary material for: Genomes comparison of two Proteus mirabilis clones showing varied swarming ability
Source: Mol Biol Rep. 2023 May 23;50(7):5817–26. doi: 10.1007/s11033-023-08518-x (PMC10290045; doi:10.1007/s11033-023-08518-x)
Supplement: Supplementary file 6 — Supplementary file6 (DOCX 17 KB) [file 11033_2023_8518_MOESM6_ESM.docx]

**Table S6** The putative antibiotic resistance genes identified in genomes of *Proteus mirabilis* isolates K38 and K39 by the Resistance Gene Identifier (RGI) based on the Comprehensive Antibiotic Research Database (CARD).

| **ARO Term*** | **SNP** | **Detection** | **AMR** | **Drug** | **Resistance** | **% Identity of Matching Region** | **% Length of Reference Sequence** |
| --- | --- | --- | --- | --- | --- | --- | --- |
|  |  | **Criteria** | **Gene Family** | **Class** | **Mechanism** |  |  |
| *adeF* |  | protein homolog model | resistance-nodulation-cell division (RND) antibiotic efflux pump | fluoroquinolone antibiotic, tetracycline antibiotic | antibiotic efflux | 42.17 | 99.34 |
| *rsmA* |  | protein homolog model | resistance-nodulation-cell division (RND) antibiotic efflux pump | fluoroquinolone antibiotic, diaminopyrimidine antibiotic, phenicol antibiotic | antibiotic efflux | 92.98 | 101.64 |
| *Klebsiella pneumoniae* KpnH |  | protein homolog model | major facilitator superfamily (MFS) antibiotic efflux pump | macrolide antibiotic, fluoroquinolone antibiotic, aminoglycoside antibiotic, carbapenem, cephalosporin, penam, peptide antibiotic, penem | antibiotic efflux | 72.71 | 100.00 |
| *catA4* |  | protein homolog model | chloramphenicol acetyltransferase (CAT) | phenicol antibiotic | antibiotic inactivation | 96.77 | 100.00 |
| *Klebsiella pneumoniae* KpnF |  | protein homolog model | major facilitator superfamily (MFS) antibiotic efflux pump | macrolide antibiotic, aminoglycoside antibiotic, cephalosporin, tetracycline antibiotic, peptide antibiotic, rifamycin antibiotic | antibiotic efflux | 68.81 | 100.92 |
| *vanG* |  | protein homolog model | glycopeptide resistance gene cluster, Van ligase | glycopeptide antibiotic | antibiotic target alteration | 39.09 | 104.87 |
| *qacJ* |  | protein homolog model | small multidrug resistance (SMR) antibiotic efflux pump | disinfecting agents and antiseptics | antibiotic efflux | 37.25 | 102.80 |
| *tet(D)* |  | protein homolog model | major facilitator superfamily (MFS) antibiotic efflux pump | tetracycline antibiotic | antibiotic efflux | 53.79 | 101.02 |
| *qacJ* |  | protein homolog model | small multidrug resistance (SMR) antibiotic efflux pump | disinfecting agents and antiseptics | antibiotic efflux | 37.86 | 100.93 |
| CRP |  | protein homolog model | resistance-nodulation-cell division (RND) antibiotic efflux pump | macrolide antibiotic, fluoroquinolone antibiotic, penam | antibiotic efflux | 98.1 | 100.00 |
| *Haemophilus influenzae* PBP3 conferring resistance to beta-lactam antibiotics | D350N | protein variant model | Penicillin-binding protein mutations conferring resistance to beta-lactam antibiotics | cephalosporin, cephamycin, penam | antibiotic target alteration | 51.45 | 98.03 |
| *Morganella morganii* gyrB conferring resistance to fluoroquinolones | S463A | protein variant model | fluoroquinolone resistant *gyrB* | fluoroquinolone antibiotic | antibiotic target alteration | 84.58 | 100.00 |
| *Escherichia coli* EF-Tu mutants conferring resistance to Pulvomycin | R234F | protein variant model | elfamycin resistant EF-Tu | elfamycin antibiotic | antibiotic target alteration | 94.66 | 96.09 |
| * ARO - Antibiotic Resistance Ontology | | | |  |  |  |  |
